# Lipids and Lipoproteins in Atherosclerosis

**DOI:** 10.3390/biomedicines11051424

**Published:** 2023-05-11

**Authors:** Evgeny Bezsonov, Victoria Khotina, Victor Glanz, Igor Sobenin, Alexander Orekhov

**Affiliations:** 1 Laboratory of Angiopathology, Institute of General Pathology and Pathophysiology, 8 Baltiiskaya Street, 125315 Moscow, Russia; nafany905@gmail.com; 2 Avtsyn Research Institute of Human Morphology of Federal State Budgetary Scientific Institution “ Petrovsky National Research Centre of Surgery”, 3 Tsyurupa Street, 117418 Moscow, Russia; 3 Department of Biology and General Genetics, I. M. Sechenov First Moscow State Medical University, 8 Izmailovsky Boulevard, 105043 Moscow, Russia; 4 Laboratory of Medical Genetics, Institute of Experimental Cardiology, National Medical Research Center of Cardiology, 15a 3rd Cherepkovskaya Street, 121552 Moscow, Russia

Atherosclerosis is a chronic inflammatory disease [1,2] which manifests in the pathological thickening of the walls of large arteries, and is related to lipid depositions. Lipids, including cholesterol and triglycerides, are transported by lipoproteins (particles mostly consisting of protein and lipids) in the blood. Different types of lipoproteins can be isolated from human blood when their density is taken into the account (for example, low-density lipoproteins (LDLs) and high-density lipoproteins (HDLs)).

Multiple pathological factors are associated with the development of atherosclerosis, including its relatively recently discovered association with mutations of mitochondrial DNA and mitochondrial dysfunction [1], which are also associated with various diseases and conditions, such as non-alcoholic fatty liver disease, diabetes, and senescence [3,4,5]. The role of non-coding RNA [6], exosomes [7], autoimmune reaction [8], sex [9], and even amyloid deposits [10] in atherosclerosis development is receiving more and more attention, leading to the application of approaches of corresponding disciplines [6,7,8,9,10,11,12,13,14] for the development of novel therapeutic strategies.

However, multiply-modified LDL (mmLDL) is classical component circulating in the blood of patients and inducing pathological changes upon atherosclerosis. mmLDL possesses the property of atherogenicity—the capability to induce the accumulation of cholesterol in cultivated cells, such as macrophages. It is an important pattern of atherosclerosis pathogenesis since native LDL is not capable to induce such an accumulation. Among the modifications of LDL which have been studied are oxidation [15], and desialylation [16]. HDL is involved in the transport of cholesterol from the arterial wall, playing an important role in regulating lipid levels with the low level of HDL that is connected with atherosclerosis development [17].

Dr. Vladimir Tertov (1955–2001) studied the role of altered lipoprotein metabolism (including discovering the existence of desialylated LDL in the blood of atherosclerotic patients and the atherogenic effects of anti-LDL antibodies) in the cellular mechanisms of atherogenesis, which resulted in the prominent discovery of the existence of trans-sialylating activity in human serum and the isolation of the corresponding protein, whose coding gene was, unfortunately, not identified [18]. Recently, it was found that *NEU1* (neuraminidase 1, possesses desialylating activity) expression is elevated upon pro-inflammatory stimulation of monocyte-like cells (it was also found to be elevated in macrophages in atherosclerotic plaques) [19]. In addition, the targeted inhibition of Neu1 and Neu3 activity led to a reduction in the size of atherosclerotic lesions in a mouse model [20]. Combined together, the aforementioned evidence suggests a strong association of desialylating activity in the blood and tissues of patients with the formation of desialylated LDL and the progression of atherosclerosis. Thus, neuraminidases (and, most probably, Neu1 and Neu3) can be considered as a new pharmacological target for anti-atherosclerotic therapy. Figure 1 illustrates the current views on the role of neuraminidases (sialydases) and LDL desialylation in the pathogenesis of this disease.

The Special Issue “Lipids and Lipoproteins in Atherosclerosis: A Commemorative Issue in Honor of Dr. Vladimir V Tertov” contains 14 high-quality papers which shall be discussed briefly below.

Strong correlations between subfractions of HDL and markers of inflammation and progranulin were found, suggesting the anti-atherogenic effect of progranulin in heterozygous familial hypercholesterolemia, possibly due to the alteration of HDL’s composition and the reduction in inflammation [21].

It has been found that intermittent hypoxic–hyperoxic exposures at rest lead to improvements to the arterial stiffness, lipid profile, and functional state of the liver in patients with metabolic syndromes, meaning that it could be used as a therapy in the treatment and prevention of atherosclerosis, obesity, and other pathologies associated with metabolic syndrome [22].

An association of systemic inflammation (detected by the high levels of high-sensitivity C-reactive protein) with atherogenic dyslipidemia related to increased levels of LDL cholesterol or an increased ratio of total cholesterol/HDL cholesterol was found for subclinical atherosclerosis in the case of individuals suffering from familial hypercholesterolemia [23].

It has also been found that mitochondrial transplantation to 7-ketocholesterol-loaded murine macrophages (cell line RAW264.7) leads to reestablished phagocytosis and a reduction in lipid content, as well as restored expression of CPT1a and anti-inflammatory cytokines. Thus, mitochondrial transplantation could be a novel therapeutic approach for the correction of foam cells in the case of atherosclerosis [24].

Statistical and bioinformatic analysis of lipid-associated loci found by genome-wide association studies was conducted in order to discover the possible mechanisms of these loci and their involvement in the pathological changes occurring due to coronary artery disease (CAD) [25]. Polymorphisms of the *APOC1*, *LPA*, *F2*, and *PLTP* genes were found to be associated with the risk of CAD, regardless of body mass index, age, or sex [25].

The influence of alirocumab (PCSK9 antibodies) treatment on the metabolism of triglycerides was studied, with no significant effects being found on the levels of fasting triglycerides, post-prandial triglycerides, or proteins related to the regulation of lipoprotein lipase [26]. The short-term influence of alirocumab on vascular function has been studied, with no significant changes observed in the thickness of the carotid intima–media, brachial artery flow-dependent dilatation, or carotid artery fractional anisotropy [27].

The association between HDL cholesterol levels and adverse cardiovascular disease outcomes in case of the presence of diabetes in patients with acute ischemic stroke was studied [28]. A linear association was found between the levels of HDL cholesterol and the risks of major adverse cardiovascular events (MACEs) and recurrent stroke. Low levels of HDL cholesterol were connected with increased risks of MACEs and recurrent stroke [28].

It was found that hyperleptinemia led to the increase of oxidized HDL levels [29]. HDLs isolated from normal-weight healthy men (nwHD) induced a reduction in pro-inflammatory cytokines’ production and marker gene expression profile characteristics for the M1 phenotype in microglial cells BV2 [29]. The co-administration of LPS and leptin with nwHDL or HDL isolated from obese men led to the activation of BV2 microglial cells, resulting in the increased production of pro-inflammatory cytokines and proving that obesity led to the reversal of the anti-inflammatory and antioxidant activities of HDLs in a microglial cell model [29].

Hypoxia and morbid obesity were shown to lead to an increase in the expression of genes of scavenging receptors (*MSR1*, *LOX-1*, *CXCL16*, and *CL-P1*), as well as markers of inflammation (*TNFα* and *IL6*) in visceral adipocytes in humans [30]. Hypoxic conditions led to the increase of the pro-inflammatory stimulation of visceral adipocytes by oxidized LDL [30].

A review was written devoted to the role of the viruses in the development of atherosclerosis, including their characteristics, potential mechanisms of the influence of viral infections on atherosclerosis progression, and the current approaches for antiviral treatments and the prevention of viral infections [31].

The current ideas and concepts related to the role of vimentin filaments in the functioning of endothelial cells and in the development of inflammation and atherosclerosis were discussed and reviewed [32].

Another review was written discussing the current situation in the field of lipid droplet dynamics in immune cells, the regulation of the metabolism, and the availability of arachidonic acid, with a focus on atherosclerosis [33].

The role of Vladimir Tertov in the study of desialylated lipoproteins, in the finding of the connection of modified (and desialylated) LDL with atherosclerosis development, and in the discovery of sialidase activity in human serum was reviewed [34].

## Figures and Tables

**Figure 1:**
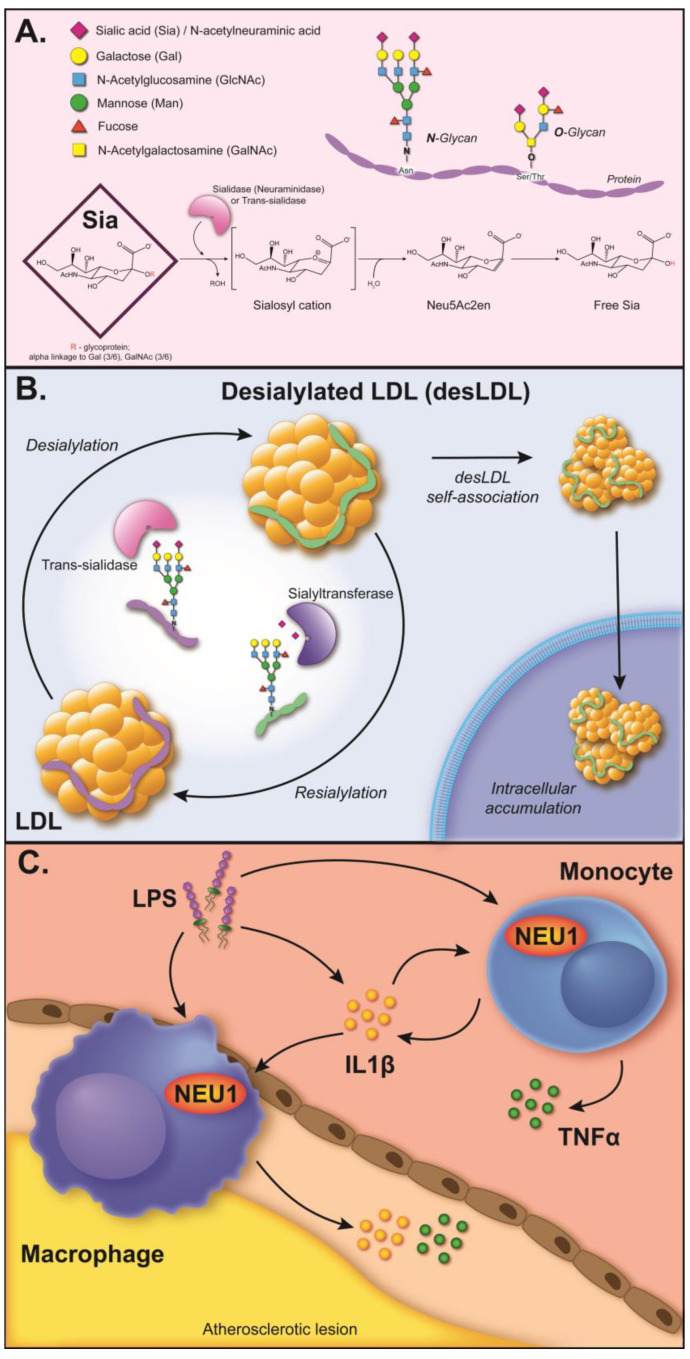
The role of LDL desialylation in atherosclerosis development. (**A**) The structure of sialic acid-containing glycosylation of protein, and the mechanism of the removal of sialic acid residue [16]. (**B**) The formation of desialylated LDL, leading to its self-association and intracellular accumulation [16]. The activity of trans-sialidase, discovered in human serum by Vladimir Tertov, could result in the generation of desialylated LDL (by using native LDL as a donor of sialic acid residue for its further transport to the acceptor molecule). The impaired activity of cellular sialyltransferases could also potentially result in reduced sialylation of LDL (in comparison with native LDL). (**C**) The mechanism of elevated expression of *NEU1* in monocytes/macrophages (including the ones located in atherosclerotic lesions) after pro-inflammatory stimulation [19]. Lipopolysaccharide (LPS) and IL1𝛽 induce elevated expression of *NEU1* in the monocyte-like THP-1 cell line. Macrophages in the intima layer, calcified regions, and the adventitia of atherosclerotic plaque also have elevated levels of Neu1 [19]. If the elevated Neu1 protein in monocytes/macrophages obtains access to native LDL, then it could become a self-perpetuating mechanism of the generation of desialylated LDL (since modified LDL can also be a trigger of pro-inflammatory activation of immune cells).
